# Atypical dependence of excited exciton energy levels and electron-hole correlation on emission energy in pyramidal InP-based quantum dots

**DOI:** 10.1038/s41598-021-04084-z

**Published:** 2022-01-07

**Authors:** Michał Gawełczyk

**Affiliations:** 1 Department of Theoretical Physics, Faculty of Fundamental Problems of Technology, Wrocław University of Science and Technology, 50-370 Wrocław, Poland; 2 Institute of Physics, Faculty of Physics, Astronomy and Informatics, Nicolaus Copernicus University in Toruń, Grudziądzka 5, 87-100 Toruń, Poland

**Keywords:** Quantum dots, Theoretical physics, Quantum dots

## Abstract

We calculate the spectrum of excited exciton states in application-relevant self-assembled pyramidal quantum dots grown in InAs/InP and InAs/AlGaInAs material systems. These types of dots have been recently shown to combine the emission in the third optical fiber window with low surface density and a reasonable level of in-plane symmetry of emitters, which predestines them for studies on single- and entangled-photon emission and for corresponding applications. The spectrum of optically active excited states is crucial for successful resonant and quasi-resonant excitation of emitters, allowing for conservation of angular momentum and addressing individual selected quantum states. Here, we show that in both types of studied dots, due to their specific morphology of truncated pyramid, the density of excited-state ladder, especially the *s–p* shell splitting may follow an unconventional dependence on emission energy, opposite to the one typically met in regular quantum dots. We obtain this result via modeling based on available morphological data and calculation within the multi-band ${{\varvec{k}} {\cdot } {\varvec{p}}}$ envelope-function theory combined with the configuration-interaction method used to calculate exciton states. Then, we explain this observation in purely geometric terms, as a result of an increasing effective quantum confinement width in a pyramid that is progressively cut from the top. Additionally, we show that the inverted trend is also manifested in the amount of electron-hole correlation in the exciton ground state, which also shows an anomalous dependence on emission energy and quantum dot volume.

## Introduction

Semiconductor quantum dots (QDs) are constantly attracting attention in various research fields, but the aspects on which the strongest emphasis is placed change from a decade to a decade. Currently, as their fundamental properties seem to be well described, such nanostructures migrate from basic research to applications, where they may be adopted in various ways and for multiple purposes. One of the most prominent features of the specific type of QDs that are made by self-assembly is their optical activity, i.e., the ability to absorb photons, which excite electrons from the valence-band to the conduction-band states, creating interacting electron-hole pairs called excitons. Such nanostructures are formed of material coming from a strained layer of one semiconductor deposited on another one with mismatched lattice constant when fragmentation of the layer into nano-islands reduces the elastic energy of the system. After covering with another layer of the first material, QDs are permanently embedded within the bulk barrier material.

While various pairs of semiconductor materials have been used to fabricate QDs, recently, there has been a particularly dynamic development of such technology for InAs embedded in matrices of InP-based alloys, namely various zincblende III–V semiconductor alloys that are lattice-matched to InP^1^. The reason is that such nanostructures emit light in the range of wavelengths coinciding with the third telecom window, defined by minimal losses for transmission of light through optical fibers, and centered on 1.55 $\upmu$m. Combining this with the quantum nature of carrier states in dots opens the possibility for application as sources of non-classical light (photon) states perfectly suited for exploitation in ultra-secure quantum communication schemes^2^ in long-haul telecom fiber networks^3^. However, single emitters are needed for this, i.e., QDs spatially and spectrally (energetically) isolated from others. At this point, a problem arises, as quantum dots manufactured in the InP-based material systems naturally grow in dense ensembles^4–7^, resulting from the relatively small lattice mismatch between the QD and barrier materials. The latter, combined with in-plane anisotropic indium diffusion, which is one of the driving factors in the growth by molecular beam epitaxy, causes such QDs to usually grow in a highly elongated shape^7–9^, which results in a dense and atypical spectrum of exciton states^10,11^ with significant fine structure splitting^12^ and specific optical properties^13^. Thus, various modified growth methods have been developed to decrease the areal density of such dots and enhance their symmetry^14–17^. Here we focus on QDs prepared using two such methods. While technically different and demonstrated for different matrix materials, they both result in similar truncated-pyramid geometry of produced QDs. The first of the methods is based on introducing an additional stage during the molecular beam epitaxy of samples, called the ripening step^18^, and has been recently successfully used to prepare low-density InAs/InP^19,20^ and InAs/AlGaInAs dots^16,21–23^ (by AlGaInAs we mean the specific commonly used Al$_{0.24}$Ga$_{0.23}$In$_{0.53}$As alloy). The ripening stage leads to redistribution of InAs, which results in smaller number of larger dots with strongly enhanced in-plane size uniformity. The second method, which is the growth using metal-organic vapor phase epitaxy^17^, is even more promising, as the transfer of this technological process to the industry should be more accessible. Also in this case, the successful growth of low-density ensembles of InAs/InP QDs has been reported^24^.

Here, we focus on the similarities of these systems, which stem from the fact that both technological processes used to prepare them include a stage at which the initially formed nano-islands are partially degraded. During this, the material, mainly from their tops, becomes mixed into the top barrier material layer. Very recently, microphotoluminescence excitation^25^ studies of such InAs/InP QDs showed that they possibly have a peculiar feature, namely, that the *s*–*p*-shell splitting for excitons in such systems may follow dependence on emission energy, which is opposite to the standard increasing one^26^. There was a single report on similar behavior of InAs/GaAs QDs with a strain-reducing layer, which was attributed to a strong variation of average In content within QD ensemble with a specific form of composition gradient within a QD^27^. Determination of such an atypical trend is practically essential, as it has to be taken into account when studies exploiting exciton excited states are performed. For instance, *p*-shell excitation scheme has been shown to lead to exceptionally high purity of single-photon emission^28^. Additionally, like any anomalous behavior, it states an interesting basic research problem. Such an inverted dependence forms a gap in, seemingly nearly complete nowadays, understanding of the physics of excitons confined in semiconductor nanostructures.

In this work, we use the available data on the growth and resultant morphology of considered quantum dots^22–24^ and use it to perform detailed theoretical modeling. With this, we find the experimentally noticed inverted trend in the calculated exciton energy levels. Based on our results, this peculiarity may be attributed solely to the geometry of dots. Namely, as a product of modified self-assembled growth processes, in which the initially formed large nano-islands of InAs have partially deteriorated after the overgrowth, such dots have the form of truncated pyramids. Contrarily to typical dots grown in GaAs matrix or in InP-based ones but made in a standard growth process, these dots are not characterized with a fixed value of the width-to-height ratio. Conversely, in this case, these two dimensions are not strictly connected. While most probably the height of initially formed pyramids is in a well-defined ratio to the base size, the truncation is then arbitrary up to some level. As a result, the emission energy, which equals the exciton ground-state energy and depends essentially on the height, i.e., the smallest dimension, is weakly related or even unrelated to the dot’s base size. As a result, the typical increasing trend of exciton *s*–*p*-shell splitting versus emission energy is absent.

Then, the emergence of the opposite one may be understood based on simple geometric facts, confirmed by our calculations. Namely, the dimension of the in-plane confinement for carriers is not directly defined by the base area of the dot but rather connected with an effective one, a good measure of which is the width averaged along the growth axis. For the typical lens-shaped QDs, these two are similar, but the difference is significant in pyramidal geometry, where the height is defined by truncation. Namely, the complete pyramid is effectively smaller in-plane than the truncated one for a given base size and inclination of sidewalls. It becomes evident when width at half height is taken as a measure of the effective width. This impact is present in the calculated wave functions and leads to the discussed inverted trend of energy level splitting.

Additionally, the inverted trend of energy splitting is reflected in other features of exciton states like the amount of electron-hole correlation in the exciton ground state due to Coulomb interaction. The latter’s significance becomes large when energy splitting is small enough to be comparable with the typical Coulomb interaction scale of $\sim ~20$ meV. Thus, also in this aspect, we deal with a reversed dependence on emission energy inherited after the shell splitting.

In the following, we first introduce the theoretical model, then present the results and finally discuss and conclude them. More technical details are given in the “Methods” section.

## System and theoretical model

**Figure 1:**
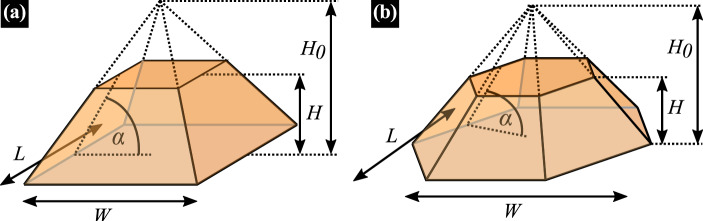
Geometry of modeled QDs. Schematic presentation of the InAs/InP (**a**) and InAs/AlGaInAs (**b**) QD geometry. Wetting layers are not shown.

We model the considered QDs based on available morphological data. Both types of studied dots grow in the form of truncated pyramids but differ in the shape of the base. Those formed by ripening in the InAs/AlGaInAs material system are nearly symmetric^16,19,21^, so the base shape is close to a square, while for the other, the base has the form of a slightly asymmetric hexagon with elongation along the $[1{\bar{1}}0]$ crystallographic direction^24^. Dots of both types, as a consequence of the Stranski–Krastanov growth mode, protrude from a thin InAs wetting layer. A schematic pictures of the simulated geometry for both types of dots are presented in Fig. 1a, b, respectively. In general, we allow for in-plane asymmetry defined by the base length *L* along $[1{\overline{1}}0]$ and width *W* along [110], as well as for varying inclination of sidewalls. According to structural data, the most typical geometry of InAs/InP QDs is given by Fig. 1a with $L\simeq W\ge 30$ nm, and $\alpha \sim 30^{\circ }$. For the InAs/AlGaInAs system (Fig. 1b) the in-plane size varies with a typical ratio of $L/W\sim 1.3$, while $\alpha \sim (25{-}30)^{\circ }$. In both cases, the height *H* varying from single InAs monolayers ($\sim 0.3$ nm) to a few nm is the main source of emission energy distribution.

**Figure 2:**
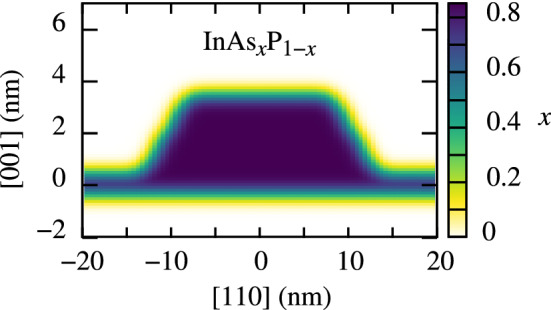
A Cross-section of an exemplary InAs/InP QD material composition. Color indicates the local *x* value, i.e., As concentration.

We study various QD compositions *c*, from clean InAs in a QD ($c=1$), up to 30% mixing with the barrier material ($c=0.7$). We initially assume that both the dot and wetting layer are made of homogeneously distributed InAs$_c$X$_{1-c}$, where X is the barrier material (InP or AlGaInAs). We then perform Gaussian averaging with $\sigma = 0.6$ nm of the three-dimensional material composition profile to simulate the interdiffusion of atoms at interfaces, which is well described by normal diffusion^29^. An exemplary resultant continuous profile of material composition (given by the value of *x* in InAs$_x$X$_{1-x}$) is shown in Fig. 2 for an InAs/InP QD with $c=0.85$.

For such material profiles, we calculate the structural strain resulting from the mismatch of lattice constants between the two materials and the resultant shear–strain induced nonuniform piezoelectric field, as the materials in question are piezoelectric. With this, eigenstates of electrons and holes are calculated within the multiband ${{\varvec{k}} {\cdot } {\varvec{p}}}$ theory. In the calculation, we also consider the influence of the piezoelectric field and the spin–orbit coupling, which have a significant impact on the states of carriers in quantum dots^30^. Finally, using the configuration-interaction method, we find the states of excitons, and the strength of their coupling to light. We discuss the calculation in more detail in the “Methods” section, where material parameters are also given in Table 1.

## Results and discussion

In this section, we present the results of our calculation, including the computed eigenstates of both types of carriers and their Coulomb-bound complexes, as well as their optical properties.

**Figure 3:**
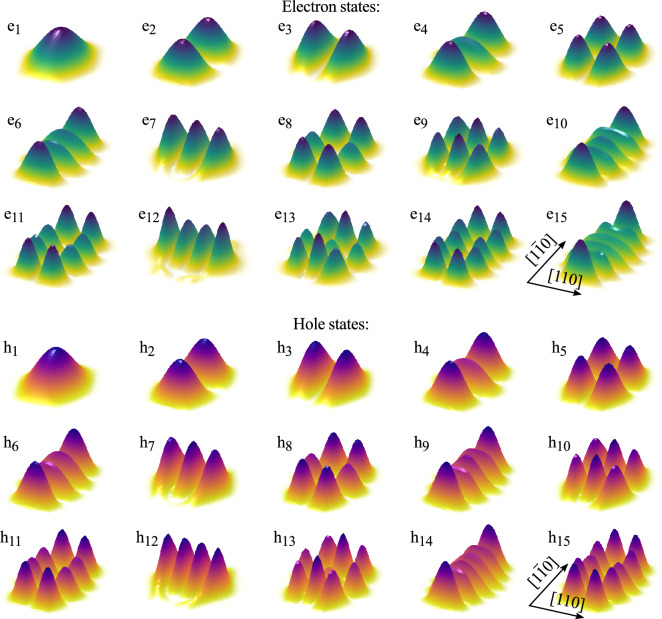
Electron (top) and hole (bottom) eigenstates. The projection, onto the (001) plane, of the probability density in a number of lowest-energy orbital eigenstates for an exemplary InAs/AlGaInAs QD with $c=1$, $L=43$ nm, $W=33$ nm, $H=2.4$ nm, and $\alpha =30^\circ$.

We have calculated a number of single-particle, i.e. electron and hole, eigenstates for each type of simulated QDs. To begin, we present in the top and bottom parts of Fig. 3 the probability densities of finding the electron and the hole, respectively, projected onto the (001) plane, $\left|\psi (x,y)\right|^2=\int _{-\infty }^{\infty }\mathrm {d}z\,\left|\psi ({\varvec{r}})\right|^2$, in an exemplary InAs/AlGaInAs QD with $c=1$, $L=43$ nm, $W=33$ nm, $H=2.4$ nm, and $\alpha =30^\circ$, in a number of lowest-energy orbital levels. One may notice the formation of 2D-like *s*, *p*, and *d*, etc., shells. The shells are affected by the broken cylindrical symmetry, as the dot is slightly in-plane elongated. This asymmetry is reflected in the partial formation of nondegenerate axis-wise excitations (e.g., for states 2 and 3 in both carrier types) instead of their rotationally symmetric equal superpositions. Note that one finds such a result even for fully symmetric geometry, as the shear strain at material interfaces and the resultant piezoelectric field are enough to lift the degeneracy^31^.

**Figure 4:**
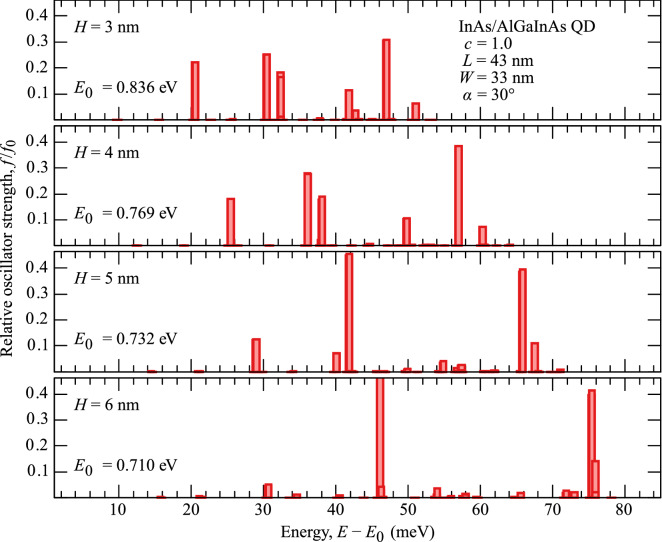
Impact of QD height on the exciton spectrum. Relative oscillator strength of exciton excited states as a function of their energy relative to the ground-state energy, plotted for varying height (consecutive panels) of an exemplary InAs/AlGaInAs QD.

Using an electron-hole configuration basis of products of single-particle eigenstates, we calculate the states of excitons. Based on the spin configuration and matching parity of electron and hole states contributing to a given exciton eigenstate, it may be bright, i.e., couple to light, or dark, when it does not couple. We calculate the respective optical transition matrix elements for a number of excited states, and in Fig. 4 present the results for the exemplary InAs/AlGaInAs QD geometry. Consecutive panels show the oscillator strength *f* for *H* from 3 to 6 nm as a function of excited-state energy measured relative to the ground state. The values of *f* are also given relative to the ground-state value, as its fraction. Thus, each bar with a reasonable height represents a single bright exciton state with a radiative lifetime $\tau$ inversely proportional to the oscillator strength, $\tau \simeq 45 (\lambda \,[\mathrm {nm}])^2/(nf)$^32^, where $\lambda \,[\mathrm {nm}]\simeq 1.2398/E\,[\mathrm {eV}]$ is the emission wavelength, and *n* is the refractive index. With increasing QD height, one may notice that the ladder of bright excited states becomes more sparse, contrary to the general expectation that it should get denser for higher QD volume. We may also focus on the first excited shell and track its shift from $\sim 20$ to $\sim 30$ meV. This is the first manifestation of the main result that we want to emphasize here: while the emission energy decreases, the relative energy of excited states undergoes an opposite change, in contrast to what is normally found in QDs.

**Figure 5:**
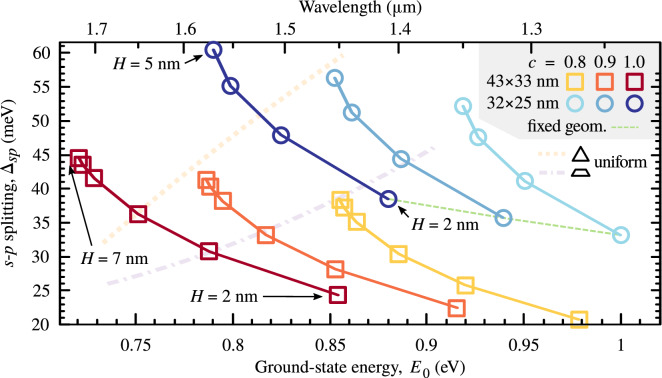
Energy splittings in InAs/AlGaInAs QDs. Dependence of the *s*–*p* shell energy splitting on the ground-state energy for InAs/AlGaInAs QDs of two base sizes (coded by the symbol type), three values of InAs concentration *c* (color), and with $\alpha = 30^\circ$. Solid lines are to guide the eye over series differing in QD height varied by 1 nm in ranges marked by labels. The dashed line connects an exemplary set of results for fixed geometry and varying *c*. Thick dotted and dash-dotted pale lines show the trends for the uniform change of size of a full and truncated pyramidal QDs, respectively.

**Figure 6:**
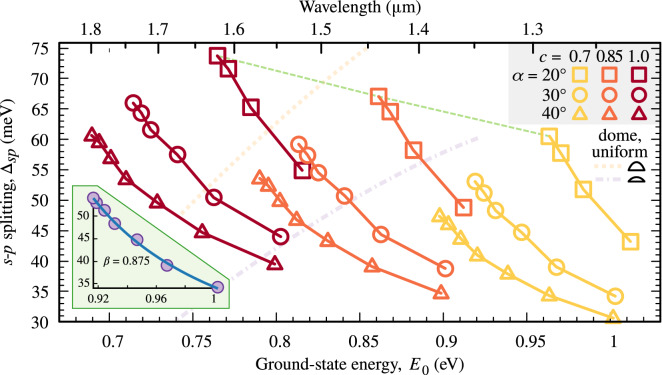
Energy splittings in InAs/InP QDs. Dependence of the *s*–*p* shell energy splitting on the ground-state energy for InAs/InP QDs with three sidewall inclination angles (coded by the symbol type), three values of InAs concentration *c* (color), and with $L = W = 30$ nm. Solid lines are to guide the eye over series differing in QD height varied by 0.6 nm starting from 1.8 nm. The dashed line connects an exemplary set of results for fixed geometry and varying *c*. Thick dotted and dash-dotted pale lines show the trends for the uniform change of size of typical dome-shaped QDs with radius to height ratios of 3 and 6, respectively. The inset shows an exemplary fit with Eq. (6).

Tracking the changes in the whole exciton spectrum is difficult to follow and quantify, so to examine the discussed inverted dependence, we focus on the splitting of the *s* and *p* shells in a QD. InP-based QDs discussed here are large in general, and with increasing QD volume, the single-particle picture breaks down in some aspects^33^. It results from the fact that single-particle configurations get mixed by the Coulomb interaction so that one cannot assign clear and unique shell labels to the eigenstates. Moreover, some moderately bright states composed mostly of nominally dark configurations emerge. Due to these issues, in the following, we use $\Delta _{sp}$ defined as the sum of electron and hole single-particle splittings. While it differs from the exact values of exciton splitting, mainly by the difference of binding energy in the ground and excited states, it may serve as a measure free from the problems mentioned above, sufficient for qualitative analysis, especially for tracking the trends and dependences.

In Fig. 5, we plot $\Delta _{sp}$ calculated for InAs/AlGaInAs QDs as a function of their emission energy. Results obtained for two QD base sizes are distinguished by the type of symbols, while the material concentration is marked with color. In total, there are six series with varying height and thus emission energy, all of which show a decreasing dependence. For comparison, with two pale lines, we also plot the trends obtained for the case of typical uniform change of size of a QD for a full (dotted line) and truncated (dash-dotted) pyramid geometry with $c=0.9$ and $\alpha =30^\circ$. In both cases, we deal with a standard increasing dependence of $\Delta _{sp}$ on ground-state energy.

Returning to the studied progressively truncated pyramid geometry, we see that similar results as above are also obtained for InAs/InP QD with a symmetric base, as shown in Fig. 6. Here, we additionally check the impact of varying QD sidewall inclination, $\alpha$, which is coded with the symbol type. As previously, color marks different QD material concentration values. Also in this case, we find the decreasing trend to be universal.

It is also informative to compare to the most common dome-shaped QDs. They are typically characterized by a fixed ratio of radius *R* to *H* for a given growth scheme. In Fig. 6, we plot with two pale lines the trends obtained for such InAs/InP QDs with $c=0.9$ and *R*/*H* equal to 3 and 6. As expected, the results follow a strong increasing trend close to proportionality, with a larger slope for less flat QDs.

From Figs. 5 and 6, we may conclude that the discussed effect is stronger, i.e. the slope of trends is larger, for smaller values of $\alpha$, thus for more inclined QD sidewalls. The other morphological parameters merely shift the whole curve horizontally or vertically.

Additionally, in both Figs. 5 and 6, we connect with dashed lines points calculated for the same height, and thus geometry of a QD but differing in material composition. We note that varying this morphological feature also yields an inverted $\Delta _{sp}$ trend, which is, however, much weaker than the one found for varying height. In real systems, these two sources may coexist, and we comment on this later.

**Figure 7:**
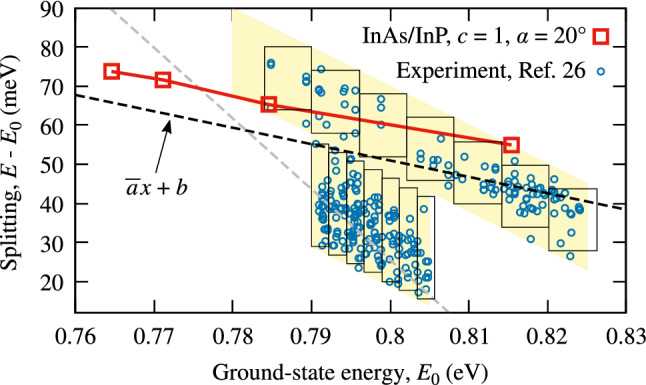
Comparison with experimental data. Circles present results from Ref.^26^ showing the energy of optically active excited states of charged excitons in InAs/InP QDs as a function of emission energy obtained in the photoluminescence excitation experiment. Squares show results of this work repeated from Fig. 6. The shaded area shows the range of energies that were accessible in the experiment. Rectangles mark subareas used here to evaluate the average slopes of the data; dashed lines show the resultant average slopes.

We may compare the above results with available experimental data. In Ref.^26^, we find the results of excited-state spectroscopy (photoluminescence excitation experiment) of InAs/InP QDs of the type discussed here. We plot these data with circles in Fig. 7. While they apparently show a decreasing trend, a careful analysis is needed here. Due to spectral filtering used in the experiment, the area of available excitation-detection energy pairs was also sloped. To estimate the actual trend in the data (or its absence) that will not be affected by this experimental condition, we divide each experimentally-available region into a series of inscribed rectangles. We fit the data with a linear function in each of them and then plot a dashed line reflecting the average of obtained slopes, $y={\overline{a}}x+b$, where ${\overline{a}}=\sum _i a_i/N$, $a_i$ is the *i*th slope and *N* is the number of rectangles. In that way, we obtain the two dashed lines for the two experimental subregions. Both show a decreasing trend.

The experiment in Ref.^26^ dealt with charged excitons with a denser energy ladder than neutral complexes. In particular, bright states below the typical *p*-shell (formed by both electron and hole in their first excited states) are present. They involve various excited hole levels and electrons in the ground state. We attribute the lower part of experimental points to transitions involving such states. Above, a typical *p*-shell state is expected, in which the transition involves an excited electron-hole pair, similarly as in the neutral exciton case. Thus, we compare the current study results with the upper part of experimental data. We find that the trend obtained here for InAs/InP QDs with $c=1$ and $\alpha ={20}^{\circ }$ (squares) fits well to the average slope of the data shown with the dashed line.

With the above observations in mind, let us try to explain the observed trends. The standard situation may be easily understood based on a simple quantum box (or anisotropic harmonic oscillator) model with confinement lengths *L*, *W*, and *H* in three orthogonal directions. In this idealized case, one gets energy levels with independent excitations along each of the directions

1 $$\begin{aligned} E_{ijk} = \hslash \omega _{L} \left( {i+\frac{1}{2}}\right) + \hslash \omega _{W} \left( {j+\frac{1}{2}}\right) + \hslash \omega _{H} \left( {k+\frac{1}{2}}\right) , \end{aligned}$$

where *i*, *j*, and *k* enumerate the excitation level for each of the axes, and the frequencies are inversely proportional to confinement lengths squared, i.e., $\omega _{L}\propto 1/L^2$, etc. Then, the ground-state energy is given for $i=j=k=0$ as

2 $$\begin{aligned} E_0=\frac{\hslash }{2}\left( {\omega _L+\omega _W+\omega _H}\right) \propto \frac{1}{L^2} + \frac{1}{W^2} + \frac{1}{H^2} \simeq \frac{1}{H^2}, \end{aligned}$$

where the last approximate equality is because $H\ll W\le L$. Thus, the ground-state energy is set mainly by QD height, i.e. the smallest dimension. On the other hand, the difference between the lowest excited state and the ground state is

3 $$\begin{aligned} \Delta _{sp} \equiv E_{100} - E_0 = \hslash \omega _L \propto \frac{1}{L^2}, \end{aligned}$$

which means that the density of the energy ladder is defined by the largest QD confinement length, so by the longer (if unequal) of the in-plane dimensions. Based on this, a common trend of changes in both energy scales is expected when the volume of a QD is increased uniformly. It is also the case in most of the experimental reports.

Here, we deal with an inversion, as the excited state splitting decreases with *H*, and thus with $E_0$. In the calculation, we change only QD height, so the first guess would be to expect approximately constant splitting, as the QD base if fixed. This is not the case, and we observe a significant decrease in splitting. It may still be fully attributed to QD geometry, but we have to consider the effective in-plane size of the confinement rather than QD base dimensions. Naively, the quantum particle experiences an effective confinement width close to the QD width at half of its height. An adequate estimation would be to average the width over the growth axis

4 $$\begin{aligned} {\widetilde{L}}(H) =\frac{1}{H} \int _0^H \mathrm {d}z\, l(z), \end{aligned}$$

where *l*(*z*) is the local QD width at given height. For the simple pyramidal geometry considered here $l(z)=L-2z/\tan \left( {\alpha }\right)$, so

5 $$\begin{aligned} {\widetilde{L}}(H) = L-\frac{H}{\tan \left( {\alpha }\right) } = \frac{L+l(H)}{2}, \end{aligned}$$

which coincides with the initial guess, and decreases linearly with *H*. A similarly monotonic, but possibly more complex, result would arise for any geometry (given the QD sidewall inclination is monotonic, i.e., the surface of the dot is convex) if the main source of varying height was the truncation of some initial/nominal QD shape from the top.

Combining Eqs. (2) and (3) with Eq. (5), we get

6 $$\begin{aligned} \Delta _{sp} \propto \left( {a - \frac{b}{\sqrt{E_0}} }\right) ^{\!-\beta }, \end{aligned}$$

where *a* and *b* are positive constants and the exponent of 2 from Eq. (3) is replaced with a parameter $\beta$, as it is known that the dependence of energy on the largest QD dimension in realistic systems is weaker than quadratic with values of $\beta$ close to 1^34^. The function fits well to the series of data from Figs. 5 and 6, and gives rather reasonable values of $\beta$. The whole data set yields average $\beta$ of $0.87\pm 0.54$. The large standard deviation reflects the approximate and idealized character of the above derivation. An exemplary fit is shown in the inset to Fig. 6.

**Figure 8:**
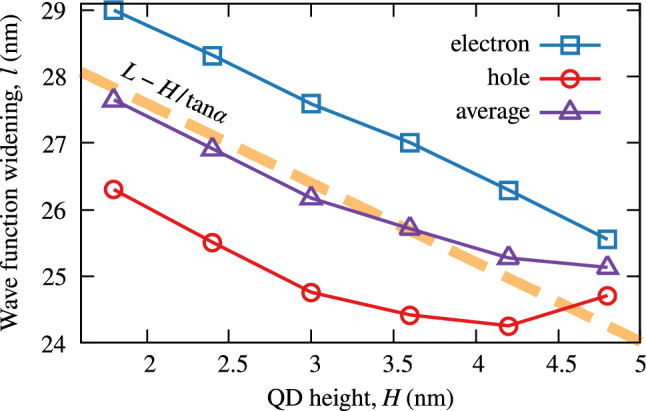
Effective QD in-plane size. In-plane extension of electron (full symbols) and hole (empty symbols) ground-state wave functions versus QD height. Solid lines are to guide the eye, while dashed line shows analytical results.

To further confirm the above reasoning, we measure the in-plane extension of electron and hole ground-state wave functions in a series of InAs/InP QDs differing in height. For this, we fit a Gaussian function to the electron density integrated over *z* axis and one of the in-plane directions and extract the widening. Note that the confinement width is related to the standard deviation of Gaussian function as $L\sim \sigma /(2\sqrt{2})$. The resultant dependence is shown in Fig. 8, where the extension of both electron and hole wave functions decreases with QD height. We also plot their average and the linear dependence from Eq. (5). The slope in the numerical data almost precisely coincides with the derived dependence in the small *H* regime. While for the electron it is the case also for higher QDs, a deviation is present for the hole, for which a nonmonotonic behavior is found. We attribute it to the fact that the large effective mass of the hole makes it more localized. For this reason, from a certain point on, the hole no longer penetrates all the available increasing QD volume. This is contrary to an implicit assumption done in the above derivation that wave-function extension follows the QD shape.

**Figure 9:**
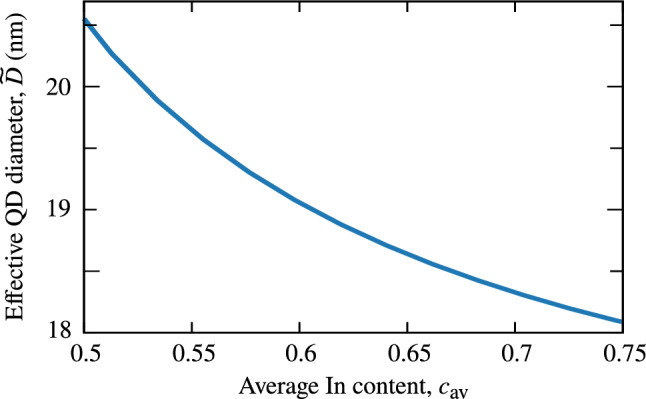
Dependence of effective QD diameter on material concentration. Effective height for InAs/GaAs QDs from Ref.^27^ as a function of average In content calculated according to Eq. (10).

We now refer to the only study known to us that considered a similar anomalous trend of *s*–*p* splitting^27^. There, InAs/GaAs QDs covered with an additional InGaAs layer introduced to reduce strain were studied. The inverted trend of $\Delta _{sp}$ on emission energy was attributed to the variation of average indium concentration among the QD ensemble, with an assumption of a specific gradient of material composition within a QD given by

7 $$\begin{aligned} c\left( {x,y,z}\right) = \left[ { c_{\mathrm {min}} + \left( {c_{\mathrm {max}} - c_{\mathrm {min}}}\right) \exp \left( { -\frac{x^2+y^2}{r_0^2} -\frac{\left( {z-z_{\mathrm {c}}}\right) ^2}{z_0^2} }\right) }\right] \,Z\left( {x,y,z}\right) , \end{aligned}$$

where $c_{\mathrm {min}}$ and $c_{\mathrm {max}}$ are the extremal concentration values, $r_0$ and $z_0$ define the in-plane and growth-axis extensions of the gradient, $z_{\mathrm {c}}$ is its *z*-axis offset, and

8 $$\begin{aligned} Z(x,y,z) = \theta \left( {z}\right) \,\theta \left( {H\exp \left( {-\frac{x^2+y^2}{r_{\mathrm {b}}^2}}\right) ^{\!\!2}-z}\right) \end{aligned}$$

determines the shape of the QD. Here, the two Heaviside functions of *z* define the flat bottom and convex top surfaces of the QD, respectively, with $r_{\mathrm {b}}$ playing the role of QD radius, and *H* being its height. Using the model of Eq. (7) with fixed $c_{\mathrm {min}}$ and varying $c_{\mathrm {max}}$, the authors obtained a desired inverted $\Delta _{sp}$ trend. The ground-state energy decreased in a standard way with increasing average In content,

9 $$\begin{aligned} c_{\mathrm {av}} = \frac{1}{V} \int \mathrm {d}^3{\varvec{r}} \,\, c\left( {x,y,z}\right) , \quad \text {where}\quad V = \int \mathrm {d}^3{\varvec{r}} \,\, Z\left( {x,y,z}\right) , \end{aligned}$$

while $\Delta _{sp}$ followed an opposite dependence.

While variation of material composition alone can also lead to such a result, as we noted discussing Figs. 5 and 6, the resultant trend is weak. We find the dependence found in Ref.^27^ to be partially a result of the composition gradient, which hides, in fact, an implicitly introduced variation of effective in-plane size of quantum confinement. When $c_{\mathrm {min}}$ is fixed and only $c_{\mathrm {max}}$ increased, we deal with a confining potential more and more concentrated in the QD center, as the potential well with inclined walls becomes deeper. This may be quantified by introducing an effective QD diameter

10 $$\begin{aligned} {\widetilde{D}} = 2 \frac{ \int \mathrm {d}^3{\varvec{r}} \, \sqrt{x^2+y^2} \, \left[ { c\left( {x,y,z}\right) - c_{\mathrm {SRL}} }\right] }{ \int \mathrm {d}^3{\varvec{r}} \, \left[ { c\left( {x,y,z}\right) - c_{\mathrm {SRL}} }\right] } \end{aligned}$$

averaged over the spatial In distribution with subtracted background of the strain reducing layer, $c_{\mathrm {SRL}}$, as the QD is not surrounded with pure GaAs. We plot the result in Fig. 9, where the effective diameter actually decreases with $c_{\mathrm {av}}$. While such a definition of ${\widetilde{D}}$ does not quantitatively reflect the resultant wave function extension in a QD, it qualitatively predicts its monotonicity.

**Figure 10:**
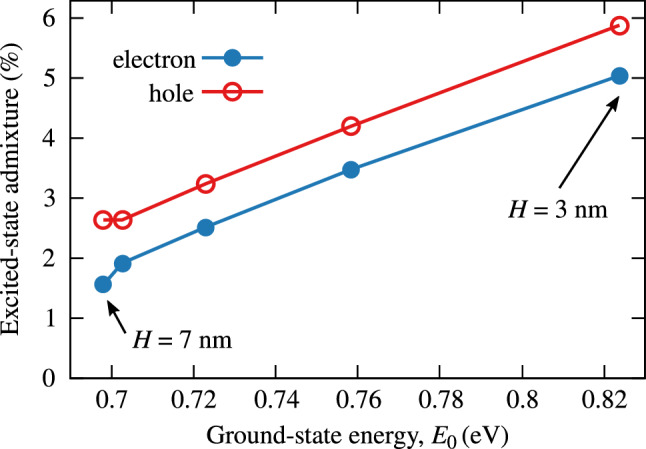
Energy dependence of electron-hole correlation. Amount of electron (full symbols) and hole (empty) excited-state admixtures to the exciton ground state plotted as a funcion of ground-state energy for InAs/AlGaInAs QDs with varying height. Lines are to guide the eye.

Having analyzed the inverted $\Delta _{sp}$ trend on emission energy and identified its reasons, we now turn to its consequences that go beyond energy shifts. In the noninteracting limit, the exciton ground state is formed solely of the ground states of the electron and the hole. This situation is modified by the fact that carriers attract each other via Coulomb interaction. In terms of single-particle electron-hole configurations, this leads to admixtures of higher configurations involving excited states of the electron, the hole, or both, to the exciton ground state. This effect is the stronger, the smaller are the shell splittings, and becomes essential when they are small enough to be comparable with the typical Coulomb interaction energy scale of $\sim 20$ meV in a QD. The amount of resultant correlations in the exciton ground state is manifested in the contribution of single-particle excited states to the exciton ground state. In Fig. 10, we plot the latter separately for the electron and the hole as a function of ground-state energy for a series of InAs/AlGaInAs QDs differing in height. As could be expected as a direct consequence of the anomalous variation of energy splittings, we also observe a trend opposite to what is typically found in QDs. We find a counterintuitive result that electron-hole pairs in QDs of smaller volume are more correlated. It is reasonable to refer to the general classification of quantum confinement strength in nanostructures into the so-called strong, intermediate, and weak confinement regimes defined originally for spherically symmetric QDs based on the relation between the dot radius and the bulk exciton Bohr radius^35^. For generic nanostructures it is adequate to use another definition formulated in terms of relationship between the Coulomb interaction energy $E_\mathrm {C}$ and electron/hole energy splittings, $\Delta _{sp}^{(\mathrm {e})}$ and $\Delta _{sp}^{(\mathrm {h})}$:

11 $$\begin{aligned} \mathrm {strong:~~~} \Delta _{sp}^{(\mathrm {e})},~\Delta _{sp}^{(\mathrm {e})}\gg E_\mathrm {C}, \quad \mathrm {intermediate:~~~} \Delta _{sp}^{(\mathrm {h})}< E_\mathrm {C} < \Delta _{sp}^{(\mathrm {e})}, \quad \mathrm {weak:~~~} \Delta _{sp}^{(\mathrm {e})},~\Delta _{sp}^{(\mathrm {e})} \ll E_\mathrm {C}. \quad \end{aligned}$$

However, it is still rather natural to identify the gradation of confinement strength with the increasing size, i.e., the volume, of the quantum dot even if its shape is far from spherical. Here, we would like to emphasize the importance of the formulation in terms of energy, as the geometric intuition fails in the presence of anomalous dependence of splittings on QD height, as discussed above, or for large QD asymmetry when a mixed confinement regime is found^36^.

## Conclusions

In conclusion, we have studied two types of quantum dots grown by self-assembly in InAs/InP and InAs/AlGaInAs semiconductor material systems. In both cases, due to the growth process aimed at reducing the in-plane density and enhancing the symmetry of nanostructures, the geometry of dots has the form of an initially large truncated pyramid. It results from partial disintegration of the initially formed pyramidal dots, as the material from the top is removed and mixed into the top barrier layer after its deposition. If two such dots differ in height, it is mainly because the different amount of material from their tops was removed, i.e., they are similar but truncated at different heights. Such a situation contrasts with size variations met typically in more standard InAs/GaAs quantum dots, or those prepared in InAs/InP-based systems but without additional efforts to assure low density.

Typically, the volume of dots grows uniformly, so if two dots from an ensemble are of different heights, their other dimensions also have approximately the same ratio. This relationship underlies the usual decreasing trend of the energy splitting between the first excited and ground exciton states versus the latter’s absolute value, which also holds for other higher-energy excited states. It results from the fact that the ground-state energy is mainly set by the smallest dimension, the dot height, while the level spacing depends on the largest, so the in-plane size. These two are typically bound by an approximately fixed ratio in standard dots. In contrast, the truncated nature of quantum dots studied here makes their height and in-plane size more independent, so dots of different height, and thus emission energy, may have bases of the same size. Additionally, if the size of the dot is not changed uniformly but by cutting off the top, the effective in-plane size experienced by carriers varies in a specific manner, given that the dot is pyramidal. Namely, the growth-axis-averaged width is a better measure of the in-plane size than the base one, as the sidewalls of the dot are inclined, and, in particular, at the top local width is zero. Thus, in such a geometry, taller dots provide effectively in-plane-narrower confinement for carriers. This fact leads to an inverted relationship between the height of the dot and the effective width of confinement it provides, and consequently, to analogously inverse scaling of level spacing compared to the ground-state energy.

We have checked this behavior for both studied classes of quantum dots and provided the splitting values between the *s* and *p* shells. Such information is vital for experiments based on quasi-resonant excitation by tuning the laser to the excited-state energy. As the spin relaxation processes in quantum dots are very slow compared to the phonon-assisted orbital relaxation, such optical pumping with polarized light may be used to inject a desired exciton spin state into the ground state. Our calculations confirmed that the energy of excited states relative to the ground state scales oppositely to the ground-state (emission) energy in considered quantum dots. Such an atypical relationship has to be taken into account when exploiting optical transitions involving higher exciton states.

Finally, we have shown that the anomalous behavior of splittings also translates into a similar inverted trend in the amount of electron-hole correlation in the exciton ground state. As a result, we get a counterintuitive result that the electron-hole pair gets less correlated when quantum dot height, and thus volume, is increased.

## Methods

**Table 1:** Material parameters used in the modeling of nanostructures and calculation of single-particle and exciton states.

Param.	Unit	AlAs	GaAs	InAs	InP	$C^\mathrm {AlAs}_\mathrm {GaAs}$	$C^\mathrm {GaAs}_\mathrm {InAs}$	$C^\mathrm {AlAs}_\mathrm {InAs}$	$C^\mathrm {InAs}_\mathrm {InP}$	Source
$a_\mathrm {}$	Å	5.66	5.65	6.06	5.87	0	0	0	0	^37^
$E_\mathrm {g}$	eV	3.1	1.519	0.417	1.42	$^{1.31x}_{-0.13}$	0.477	0.7	0.1	^37^
$E_\mathrm {p} {}^{\dagger }$	eV	19.15	23.8	21.5	20.7	0	0	0	0	^38^
$m_\mathrm {e}^{*}$		0.15	0.067	0.0229	0.0803	0	0.0091	0.049	0	^39^
$\Delta _\mathrm {}$	eV	0.28	0.341	0.39	0.11	0	0.15	0.15	0.16	^37^
$\gamma _\mathrm {1}$		3.76	6.98	20.4	4.95	0	0	0	0	^37,39^
$\gamma _\mathrm {2}$		0.82	2.06	8.3	1.65	0	0	0	0	^37,39^
$\gamma _\mathrm {3}$		1.42	2.93	9.1	2.35	0	0	0	0	^37,39^
$e_\mathrm {14}$	${{\mathrm {C}}{\mathrm {m}^2}}$	$-0.055$	$-0.205$	$-0.111$	0.016	0	0	0	0	^40^
$B_\mathrm {114}$	${{\mathrm {C}}{\mathrm {m}^2}}$	$-1.61$	$-0.99$	$-1.17$	$-1.54$	0	0	0	0	^40^
$B_\mathrm {124}$	${{\mathrm {C}}{\mathrm {m}^2}}$	$-2.59$	$-3.21$	$-4.31$	$-3.62$	0	0	0	0	^40^
$B_\mathrm {156}$	${{\mathrm {C}}{\mathrm {m}^2}}$	$-1.32$	$-1.28$	$-0.46$	$-1.02$	0	0	0	0	^40^
$C_\mathrm {k}$	eV Å	0.002	$-0.0034$	$-0.0112$	$-0.0144$	0	0	0	0	^39^
$a_\mathrm {c}$	eV	$-5.64$	$-7.17$	$-5.08$	$-6$	0	2.61	$-1.4$	0	^37^
$a_\mathrm {v}$	eV	2.47	1.16	1	0.6	0	0	0	0	^37^
$b_\mathrm {v}$	eV	$-2.3$	$-2$	$-1.8$	$-2$	0	0	0	0	^37^
$d_\mathrm {v}$	eV	$-3.4$	$-4.8$	$-3.6$	$-5$	0	0	0	0	^37^
$c_\mathrm {11}$	GPa	1250	1211	833	1011	0	0	0	0	^37^
$c_\mathrm {12}$	GPa	534	548	453	561	0	0	0	0	^37^
$c_\mathrm {44}$	GPa	542	600	396	456	0	0	0	0	^37^
$\varepsilon _\mathrm {r}$		10.06	12.4	14.6	12.4	0	0	0	0	^39^

$^{\dagger }$Values used for optical properties; for the ${{\varvec{k}} {\cdot } {\varvec{p}}}$ Hamiltonian $E_{P} = ({m_{0}}/{m_\mathrm {e}^{*}} - 1) {E_\mathrm {g}(E_\mathrm {g}+\Delta )}/{(E_\mathrm {g}+2\Delta /3)}$ was used to preserve ellipticity of the equation system for envelope functions^41,42^.

Simulated QDs were represented on an axis-wise uniform numerical grid of the local material composition values. In all numerical calculations of electron eigenstates, we use the implementation^43,44^ of the eight-band ${{\varvec{k}} {\cdot } {\varvec{p}}}$ method^45–47^. It includes effects caused by the spin-orbit interaction^39,44^, structural strain due to mismatch of lattice constant of the two materials forming the heterostructure, accounted for within the continuum elasticity theory^48,49^, as well as the resultant piezoelectric field with terms in piezoelectric polarization up to second order in strain-tensor elements^40,50^. Details of modeling^34^, and the explicit form of the Hamiltonian^51^ may be found elsewhere, while material parameters used in calculations are given in Table 1.

Next, time-reversal operation has been applied to the valence-band electron eigenstates to obtain hole states. Neutral exciton states were found within the configuration-interaction method by diagonalizing Coulomb and phenomenological anisotropic electron-hole exchange interactions expressed in the electron-hole (product) configuration basis. Optical properties of exciton states were found in the dipole approximation via calculation of their optical-transition dipole moment^52^ and resultant radiative lifetimes^53^.

## Data Availability

The datasets generated during and/or analyzed during the current study are available from the author on reasonable request.
